# Feasibility and Clinical Outcomes of Peripheral Drug-Coated Balloon in High-Risk Patients with Femoropopliteal Disease

**DOI:** 10.1371/journal.pone.0143658

**Published:** 2015-11-24

**Authors:** Shih-Jung Jang, Chien-An Hsieh, Hsuan-Li Huang, Jyh-Ming Jimmy Juang, Hsin-Hua Chou, Chueh-Yung Tsao, Tien-Yu Wu, Yu-Lin Ko

**Affiliations:** 1 Division of Cardiology, Department of Internal Medicine, Taipei Tzuchi Hospital, The Buddhist Tzuchi Medical Foundation, Taipei, Taiwan; 2 School of Medicine, Tzuchi University, Hualien, Taiwan; 3 Cardiovascular Center and Division of Cardiology, Department of Internal Medicine, National Taiwan University Hospital and National Taiwan University College of Medicine, Taipei, Taiwan; 4 Department of Industrial and Business Management, Chang Gang University, Taoyuan, Taiwan; Osaka University Graduate School of Medicine, JAPAN

## Abstract

**Background:**

Clinical outcomes of the drug-coated balloon (DCB) procedure in high-risk patients with femoropopliteal (FP) disease have not been investigated sufficiently.

**Methods:**

This retrospective, single-center study analyzed 87 patients (39% dialysis) and 97 affected legs (64% critical limb ischemia [CLI]) that underwent DCB for symptomatic FP disease from March 2013 to September 2014. Risk stratification was based on FeDCLIP (female, diabetes, dialysis, CLI, lesion length >150 mm and poor runoff) score. The DCB outcomes among the different risk groups were compared and factors predicting restenosis were analyzed during follow-up.

**Results:**

Most of study participants (84%) were moderate to high-risk patients. The procedural success rate was 100% and the 30-day major adverse vascular event rate was 2.1%. The mean lesion length was 178 ± 106 mm and the mean follow-up time was 428 ± 145 (range 50–782) days. The binary restenosis-free and clinically driven target lesion revascularization (CD-TLR)-free rates at 12 months were 77.5% and 84.3%, respectively, for all participants. No significant differences were observed in 1-year binary restenosis and CD-TLR rates in the low-, moderate-, and high-risk groups (60%, 84%, and 73%: p = 0.396; 78%, 89%, and 80%: p = 0.635, respectively). In multivariate analysis, lesion length >150 mm (Hazard ratio [HR]: 8.00, 95% confidence interval (CI) 1.12 to 55.6, p = 0.038) and Rutherford class 6 (HR: 7.09, 95% CI, 1.15 to 43.5, p = 0.034) were identified as independent predictors of binary restenosis.

**Conclusions:**

Despite general comorbidities and advanced limb ischemia, 1-year outcomes of DCB in high-risk patients with FP disease were effective. The DCB procedure holds promise to improve vessel patency; however, lesion length >150 mm and major tissue loss were independent predictors for binary restenosis after the treatment.

## Introduction

Endovascular therapy (EVT) is considered as the first treatment of choice for peripheral artery disease of variable severity [1]. In particular, angioplasty followed by nitinol stent implantation is useful in the treatment of Trans-Atlantic Intersociety Consensus (TASC) II A/B femoropopliteal (FP) artery disease, with a high initial success rate and a low fracture rate (4%) [2–4]. The main drawback of this strategy is a high restenosis rate in longer lesions, ranging from 40% to 60% at 12 months [5–7]. Furthermore, stent fracture is another important concern, especially for longer lesions. The incidence of stent fracture has been shown to increase with longer lesion length (LL), and major stent fractures have been associated with restenosis or reocclusion [8]. Currently available devices have a high restenosis rate when applied to FP lesions >15 cm [6, 7]. Drug-coated balloons (DCBs) have significantly reduced late lumen loss and clinically driven target lesion revascularization (CD-TLR) rates in TASC II A/B lesions [9–12]. Thus far, just one study has reported the efficacy of DCB in treating long FP lesions [13]. Information regarding the results of DCBs in high-risk patients with FP disease is scarce. The aim of this study was to investigate the efficacy and outcomes of the DCB angioplasty in high-risk patients with FP disease, as well as, potential predictors of binary restenosis during a 2-year follow-up.

## Materials and Methods

This study was approved by the Taipei Tzu Chi Hospital, the Buddhist Tzu Chi Medical Foundation Institutional Review Board on December 15, 2014. The constitution and operation of this review board are in accordance with the International Council for Harmonization’s Good Clinical Practices (ICH-GCP) guidelines. The protocol number of this study is 03-X27-098.

### Study population

This was a retrospective, single-center cross-sectional study, which included patients registered in a prospectively maintained database (TRENDPAD Tzuchi Registry of ENDovascular Intervention for Peripheral Artery Disease). From March 2013 to September 2014, a total of 288 patients (316 limbs) in this registry underwent EVT for FP disease. Patients who underwent DCB therapy were eligible for enrollment. The angiographic inclusion criteria included *de novo*, restenotic, in-stent stenotic, or occlusive FP lesions. Concomitant interventions for iliac or tibial lesions were allowed. After the EVT, patients were required to have either a pre-existing or re-established adequate runoff vessel with evidence of at least 1 patent crural vessel to the foot.

**Fig 1:**
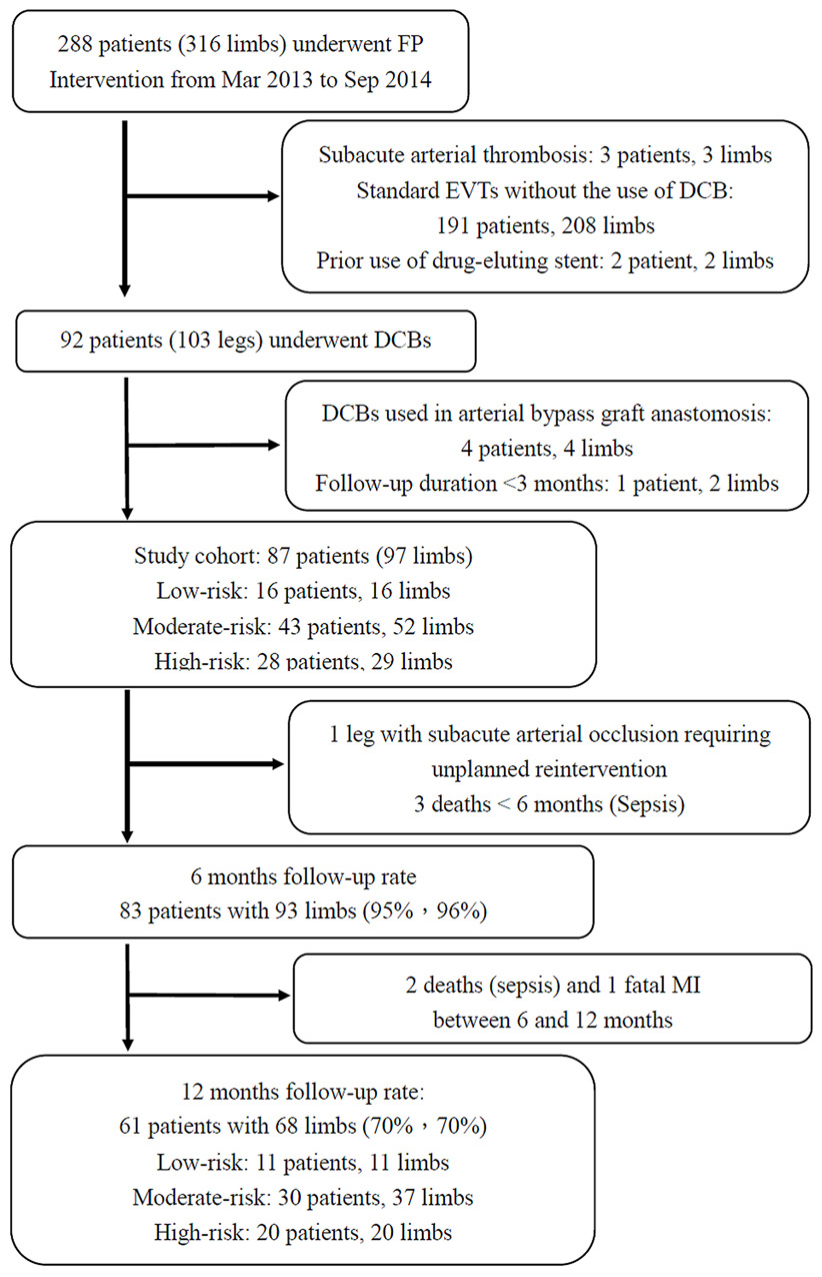
Flow chart of study participants. Flow chart of study participants. FP: femoropopliteal; DCB: drug-coated balloon; EVT: endovascular therapy; MI: myocardial infarction.

Exclusion criteria were standard EVT without the use of DCB, acute or subacute thrombotic occlusions, prior use of a drug eluting stent, prior bypass graft anastomosis lesions, a life expectancy less than 12 months, contraindications for aspirin or clopidogrel, life-threatening infections, a follow-up duration less than 3 months in surviving patients, and refusal to participate. Finally, 87 patients with 97 affected limbs were eligible for inclusion in this study. A flow-chart of the study enrollment, follow-up, compliance and analysis process is depicted in Fig 1.

All patients were informed of the risks and benefits of the DCB procedure and provided their written consent to participate prior to enrollment. The pre-interventional study comprised a clinical examination; hemodynamic evaluation (ankle or toe pressure, and duplex ultrasound [DUS]); and anatomic assessment, including computed tomographic (CT) angiography, magnetic resonance angiography, or diagnostic angiography. Toe pressures and Doppler waveform patterns were obtained to measure hemodynamic changes in patients with falsely elevated ankle brachial index (ABI) values. Demographic and interventional data, including clinical presentation according to the Rutherford classification (RC), lesion anatomy based on the TASC II system and follow-up ABIs, toe pressures, and DUS were recorded for each patient.

### Procedures

IN.PACT Admiral DCBs (Medtronic Ireland, Galway, Ireland) were used exclusively in this study. The 40 to 150-mm long balloons were coated with paclitaxel at a dose of 3 μg/mm^2^ of the balloon surface in a specific surface matrix coating consisting of urea (FreePac; Medtronic Ireland). All patients received 100 mg aspirin and 300 mg clopidogrel before the EVT. Unfractionated heparin (5,000–10,000 units) was administered during the procedure to maintain an activated coagulation time around 250 s. After crossing the lesions with a guidewire, pre-dilatation with an undersized, shorter uncoated balloon (0.5–1.0 mm smaller) was performed, followed by insertion of a DCB of appropriate size and length (a balloon/vessel diameter ratio of 1:1). In cases with LLs more than 10 cm, ≥ 2 DCBs were used with a minimum 5-mm balloon overlap at the edges. The inflation time of DCBs was 180 s using nominal pressure to allow full drug elution. Self-expanding nitinol stents were implanted in cases with a suboptimal angiographic result or flow-limiting dissection, determined by residual stenosis > 50%, trans-lesion pressure gradient ≥ 20 mmHg, or significant change in the Doppler waveform spectrum.

Intervention was performed using the angiographic imaging system (Phillips Allura Xper FD 20/10, Best, The Netherlands) and quantitative vascular angiography (Pie Medical Imaging B.V. Maastricht, The Netherlands) were performed in at least 2 orthogonal views at baseline and after the intervention A radiopaque ruler was used to calibrate angiographic measurements, including the length and minimal luminal diameter (MLD) of the target lesion and the mean proximal and distal reference vessel diameter (RVD). The percent diameter stenosis (%DS) was calculated [%DS = (1 − MLD/RVD) × 100] at baseline and after the intervention. Following the EVT, aspirin was continued indefinitely in all patients and clopidogrel was used for 3 months.

### Definitions

Procedural success was defined as the ability to successfully perform DCB angioplasty with residual stenosis ≤ 30%, with or without provisional stenting.

Binary restenosis was defined as DS > 50% by angiography or peak systolic velocity ratio > 2.5 as determined by DUS.

CD-TLR was defined as reintervention performed for > 50% DS within 5 mm of the target lesion after documentation of recurrent clinical symptoms following the index procedure.

Risk stratification was based on the FeDCLIP (female, dialysis, critical limb ischemia (CLI), LL >150 mm and poor runoff) score. The FeDCLIP score has been useful for vessel patency and mortality risk stratification after superficial femoral artery EVT [14]. A lesion length >150 mm was scored as 2 points, while female, diabetes, dialysis, CLI and poor runoff were each scored as 1 point. Scores of 0 to 2, 3 to 4, and ≥ 5 points were classified as low-, moderate- and high-risk groups, respectively.

Major adverse vascular events (MAVEs) were defined as any death, myocardial infarction (MI), unplanned reintervention, target limb amputation, or emergent surgery within 30 days.

Sustained clinical success (SCS) was defined as clinical improvement without major amputation and target leg revascularization. Event-free survival (EVS) was defined as freedom from death, MI, stroke, CD-TLR, and major amputation.

### Study outcomes measurement

The primary study endpoints were binary restenosis at 12 months in each risk group and the secondary endpoints were CD-TLR-free, SCS, and EVS rates at 12 months.

### Patient follow-up

At intervals of 1 week, 1 month, and every 3 months after EVT, each patient was assessed based on symptoms, ankle or toe brachial pressure index, and DUS. Patients with tissue loss underwent wound care, and hyperbaric oxygen therapy, by a plastic or orthopedic surgeon, until their wounds healed. Reintervention was conducted if any of the following occurred: recurrent symptoms, significant vessel stenosis (≥ 70%) with dampened Doppler waveform patterns on DUS, and an ABI decrease of ≥ 0.15. Major events (mortality, limb amputation, failure of SCS, and CD-TLR) were documented at the time of discharge or at the follow-up office visits. The alternate data sources used when office follow-up was not feasible were telephone interviews, data from medical records, local electronic medical databases, and referring physicians.

### Statistical analysis

Categorical variables are reported as counts and percentages, and continuous variables are reported as means ± standard deviation. A one-way ANOVA test was used to compare differences among the risk groups. The rates of SCS, EVS, and freedom from binary restenosis and CD-TLR for all participants and in subgroup analysis were assessed using Kaplan-Meier curves and compared with the log-rank test. Multivariate analysis was performed using Cox proportional hazards regression, entering clinically (age, gender, diabetes mellitus, hypertension, coronary artery disease, congestive heart failure, cerebrovascular accident, dialysis dependence, smoking, hyperlipidemia, Rutherford class) and anatomically (vessel calcification, occlusion, poor runoff, bailout stenting, LL >150 mm) important variables to determine the independent predictors for binary restenosis. All statistical analyses were performed with the SPSS statistical package for Windows version 21.0 (SPSS, Chicago, IL, USA). A probability value of < 0.05 was considered statistically significant.

## Results

### Baseline demographics

Table 1 summarizes the baseline demographics of the study participants. Half of them were male (51%), with a mean age of 70 ± 12 years. Patients with regular dialysis comprised 39% of study participants and 64% of affected legs had CLI. Eighty-four percent of the study participants were in the moderate to high-risk groups based on FeDCLIP scores. Significant differences in clinical and lesional factors were found among the different risk groups. Of the 97 legs evaluated, 7 iliac and 75 tibial interventions were performed during the same procedure. The mean ABIs of the target extremity at presentation were 0.52 ± 0.16 after excluding stiff arteries.

**Table 1:** Patient Demographics.

Patient Numbers:		N = 87		
Men		44 (51%)		
Age		70 ± 12 years old (33–91)		
Underlying medical comorbidities				
Diabetes Mellitus		71 (82%)		
Hypertension		72 (86%)		
Chronic renal failure		52 (61%)		
Chronic kidney disease		19 (22%)		
Dialysis dependence		33 (39%)		
Hyperlipidemia		54 (63%)		
Coronary artery disease		49 (57%)		
Smoking		35 (41%)		
Cerebrovascular accident		20 (24%)		
Congestive heart failure		14 (16%)		
Treated extremities		N = 97		
Claudication		35 (36%)		
Rest pain		14 (14%)		
Ulcer		38 (39%)		
Gangrene		10 (11%)		
Stiff artery with ABI ≥ 1.3		9 (9%)		
FeDCLIP risk scores:				
	Low-risk	Moderate-risk	High-risk	P value
Patient numbers	16	43	28	
Female	2 (12%)	20 (47%)	21 (75%)	<0.001
Dialysis	1 (6%)	13 (30%)	18 (64%)	0.001
Leg numbers	16	52	29	
LL > 150mm	2 (13%)	34 (65%)	29 (100%)	<0.001
Poor runoff^$^	11 (69%)	50 (96%)	28 (97%)	0.001
CLI	7 (44%)	30 (58%)	25 (86%)	0.006
Mean LL (mm)	106 ± 49	185 ± 108	206 ± 110	0.007
DCB length (mm)	136 ± 47	215 ± 119	245 ± 120	0.008
Target extremity ABI (all patients)		0.62 ± 0.39		
Target extremity ABI (ABI ≥ 1.3)*		0.52 ± 0.16		

Abbreviations: ABI: ankle brachial index; FeDCLIP: female, dialysis, critical limb ischemia, lesion length > 150 mm, poor runoff; LL: lesion length; DCB: drug coated balloon.

^$^ poor runoff was defined as one vessel or none of below-the-knee runoff

* Calculated by excluding ABI ≥ 1.3

### Lesion characteristics

Table 2 lists the lesion characteristics of affected legs. Of the 97 legs, 59 *de novo*, 22 restenosis, and 16 in-stent restenosis (ISR) lesions were noted. Forty-two legs (43%) had chronic total occlusion. Of the 81 *de novo* or restenotic vessels, 32 (40%) were TASC II B lesions, 28 were TASC II C lesions (35%), and 21 were TASC II D lesions (25%). Among the 16 ISR vessels, 11 Tosaka type II ISR [15] and 5 type III in-stent occlusions were noted. One-third of the affected vessels had severe calcification. Lesions located in the popliteal artery and distal portion of SFA were the most common sites treated with DCB.

**Table 2:** Lesion Characteristics.

Treated limbs: N = 97	Stenosis	Occlusion
*De novo* lesion: 59 (61%)	34	25
Restenotic lesion: 22 (22%)	10	12
In-stent lesion: 16 (16%)	11	5
Total occlusion: 42 (43%)		
Subintimal crossing: 6 (6%)		
Concomitant intervention		
Iliac intervention: 7		
Tibial intervention: 75		
Patency of below-the-knee vessels		
≤ 1 vessel run off: 76 (78%)		
≥ 2 vessel run off: 21 (22%)		
TASC classification for Non-ISR lesions: 81		
B: 32 (40%), C: 28 (35%), D: 21 (25%)		
In-stent restenosis (Tosaka class): 16		
ISR type II: 11 ISR type III: 5		
Lesion Calcification:		
Mild: 28 (29%), Moderate: 39 (40%)	Severe: 30 (31%)	
Location of DCBs: (N = 186)		
Common femoral artery: 6		
Superficial femoral artery: Proximal: 34	Middle: 34	Distal: 56
Popliteal artery: 56		

Abbreviations: TASC: Trans-Atlantic Intersociety Consensus; ISR: in-stent restenosis; DCB: drug-coated balloon.

### Immediate procedural characteristics

Table 3 summarizes the procedural results after EVT. The mean LL and the mean DCB length were 174 ± 106 and 210 ± 116 mm, respectively. For different lesion characteristics, the mean LL and the mean DCB length were as follows: 170 ± 95 and 203 ± 109 mm for *de novo* lesions, 151 ± 91 and 181 ± 90 mm for restenosis lesions, and 253 ± 129 and 279 ± 148 mm for ISR lesions, respectively. The mean DCB size and numbers in EVT were 5.23 ± 0.78 mm and 1.8 ± 1.1, respectively. Several adjuvant procedures using various devices were performed to optimize the lesions for the use of DCB, including 5 TurboHawk atherectomies (eV3, Irvine, CA, USA), 3 excimer laser angioplasties (Spectranetics, Colorado Springs, CO, USA), 2 rotational atherectomies (Boston Scientific, Natick, MA, USA), 2 peripheral cutting balloons (Boston Scientific), 1 TruePath (Boston Scientific) and 6 IVUSs (Visions^®^ PV catheter, Volcano Therapeutics, Rancho Cordova, CA, USA). Provisional stents were implanted in 24 (30%) of 81 non-ISR lesions (mean stent length 107 ± 59 mm) with either flow-limiting dissection or a suboptimal result. One patient received urgent percutaneous coronary intervention due to acute MI after EVT while another patient underwent unplanned reintervention because of sub-acute arterial occlusion 1 week after the index procedure. IVUS revealed that inadequate subintimal space without bailout stenting resulted in flow reduction and subsequent arterial occlusion. The 30-day MAVE rate was 2.1%.

**Table 3:** Immediate Procedural Characteristics.

	SFA	Popliteal artery	
Before Intervention			
RVD (mm)	5.10 ± 0.83	4.62 ± 0.78	
MLD (mm)	0.97 ± 0.91	0.76 ± 0.76	
DS (%)	81 ± 17	83 ± 17	
After Intervention			
RVD (mm)	5.29 ± 0.80	4.84 ± 0.74	
MLD (mm)	4.31 ± 0.84	3.88 ± 0.76	
DS (%)	18 ± 8	19 ± 8	
Mean lesion length (mm)	174 ± 106		
Mean DCB length (mm)	210 ± 116		
Mean DCB size (mm)	5.23 ± 0.78		
Mean DCB numbers/per leg	1.8 ± 1.1		
Lesion type	*De novo*	Restenotic	In-stent
Lesion length (mm)	170 ± 95	151 ± 91	253 ± 129
DCB length (mm)	203 ± 109	181 ± 90	279 ± 148
**Adjuvant devices**			
TurboHawk Atherectomy 5	Excimer laser 3	Rotablator 2	
Cutting balloon 2	TruePath 1	IVUS 6	
**Additional bailout stent**			
24 in 81 non-ISR lesions (30%), mean stent length: 107 ± 59 mm			
In-hospital and 30 day MAVE: 2 (2.1%)			
Myocardial infarction: 1			
Sub-acute arterial occlusion & unplanned reintervention: 1			

Abbreviation: SFA: superficial femoral artery, RVD: reference vessel diameter, DS: diameter stenosis, MLD: minimal lumen diameter, DCB: drug coated balloon, IVUS: intravascular ultrasound, ISR: in-stent restenosis, MAVE: major adverse vascular event.

### Follow-up outcomes

Over a mean follow-up of 428 ± 145 days (range 50–782), 7 patients died. Causes of death included sepsis with multi-organ failure in 6 patients and 1 fatal MI 202 days following the index procedure. Six minor amputations were performed to achieve complete wound closure and 1 major amputation was performed at 399 days after DCB treatment because of severe wound infection. One patient suffered from nonfatal MI and another patient had an ischemic stroke during the follow-up. Therefore, the EVS rate at 12 months was 74.2% for all participants but no significant differences were observed in the low-, moderate-, and high-risk groups (81.3%, 77.4% and 64.3%, p = 0.454, respectively).

Most of the study participants showed sustained clinical improvement, and follow-up ABI levels at 6 and 12 months were 0.98 ± 0.18 and 0.93 ± 0.17, respectively. Five legs required target leg reintervention due to below-the-knee restenosis instead of FP restenosis; the cumulative SCS rates at 1 year was 72.2% for all patients and there were no differences in the low-, moderate-, and high-risk groups (81.3%, 71.7% and 67.9%, p = 0.738, respectively).

**Fig 2:**
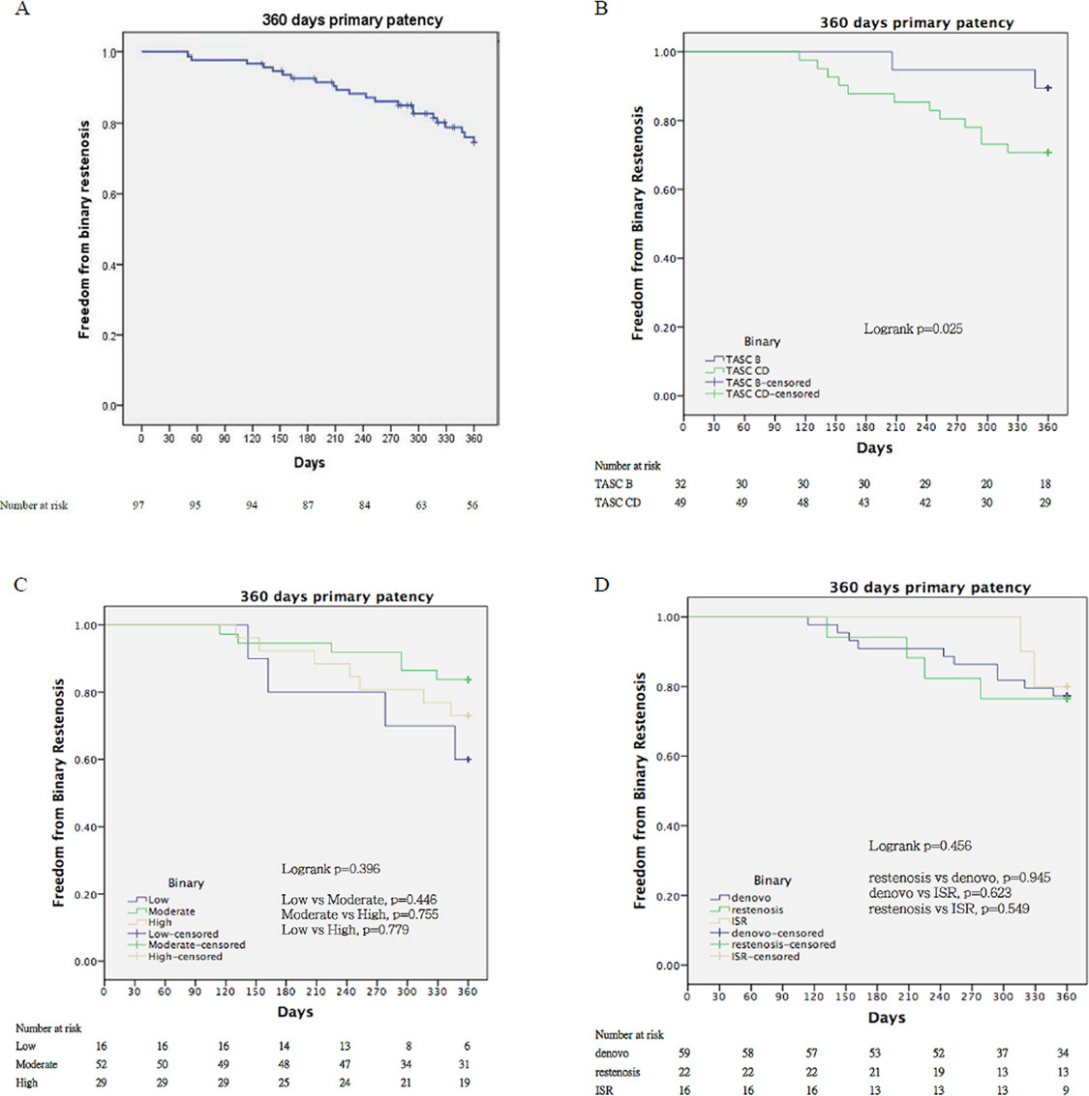
Kaplan-Meier curves for freedom from binary restenosis. (A) The 12-month binary restenosis-free rate is 77.5% for study participants. (B) A significant difference is noted between Trans-Atlantic Intersociety Consensus (TASC) II B (blue) and TASC C/D (green) lesions in the 12-month binary restenosis-free rate (90% *vs*. 71%, p = 0.025). (C) No significant differences are observed among the low- (blue), moderate- (gold), and high-risk (green) groups regarding the 12-month binary restenosis-free rates (60%, 84%, and 73%, p = 0.396). (D) The *de novo* (blue), restenosis (green), and in-stent restenosis (ISR) (gold) lesions have similar 12-month binary restenosis-free rates (79%, 75%, and 80%, p = 0.456).

**Fig 3:**
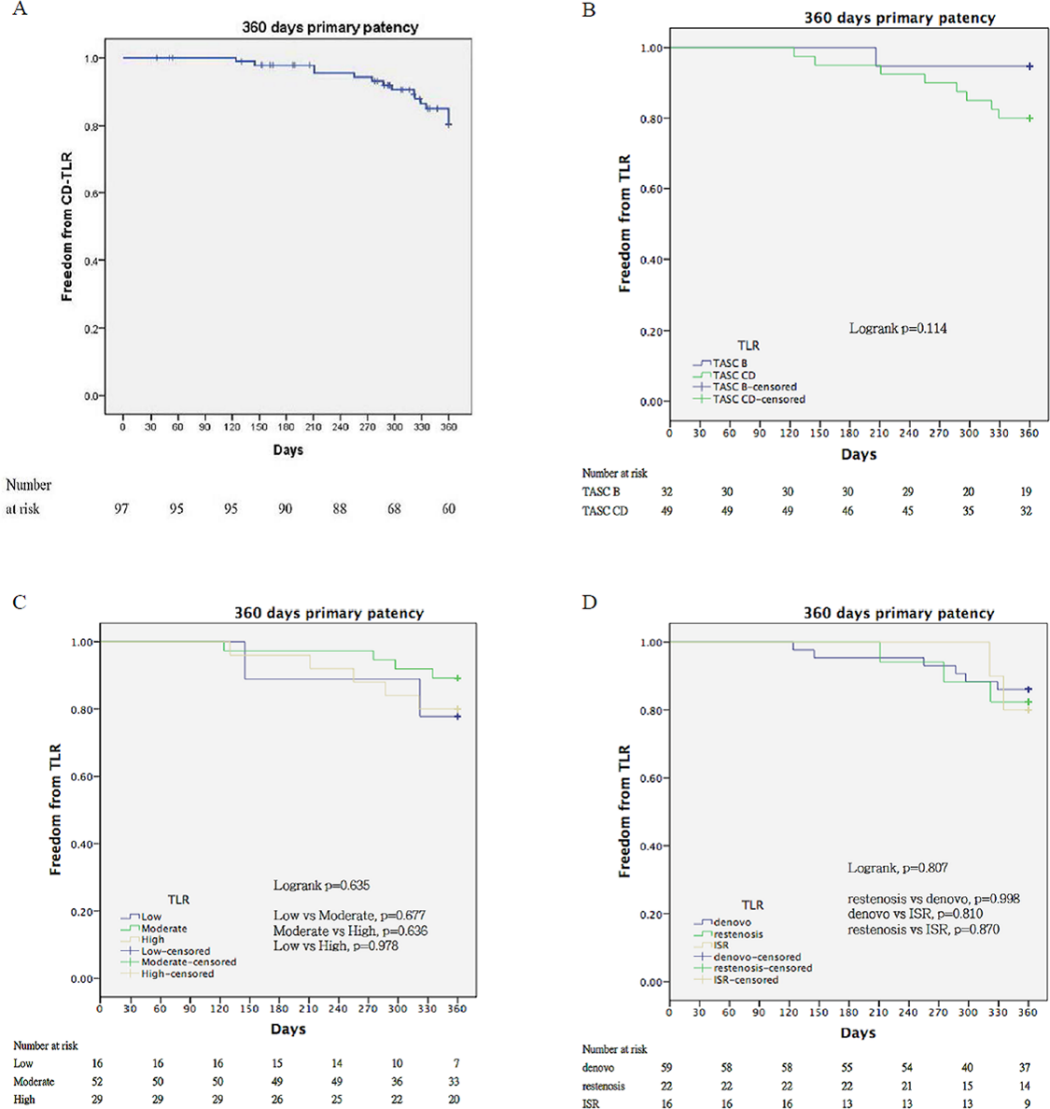
Kaplan-Meier curves for freedom from clinically driven target lesion revascularization (CD-TLR) (A) The 12-month CD-TLR-free rate is 84.5% for study participants. (B) Compared to TASC II C/D lesions (green), TASC II B lesions (blue) have a higher 12-month CD-TLR-free rate (95% *vs*. 80%, p = 0.114). (C) No significant differences are observed among the low- (blue), moderate- (gold), and high-risk (green) groups in the 12-month CD-TLR-free rates (78%, 89%, and 80%, p = 0.635). (D) The *de novo* (blue), restenosis (green), and in-stent restenosis (ISR) (gold) lesions have similar 12-month CD-TLR-free rates (80%, 75%, and 80%, p = 0.807).

The 12-month binary restenosis-free (Fig 2A) and CD-TLR-free rates (Fig 3A) for all participants were 77.5% and 84.3%, respectively. For subgroup analysis, the binary restenosis-free rate at 12 months for the TASC II B lesions (mean LL 99.3 ± 24.6 mm) was significantly better than that for TASC II C/D lesions (mean LL 213 ± 103 mm) (90% *vs*. 71%, p = 0.025) (Fig 2B). The TASC II B lesions showed a higher 12-month CD-TLR-free rate than the TASC II C/D lesions (95% *vs*. 80%, p = 0.114) (Fig 3B). There were no significant differences between the low-, moderate- or high-risk groups in terms of binary restenosis-free rate (60%, 84%, and 73%, p = 0.396) (Fig 2C) or CD-TLR-free rate (78%, 89%, and 80%, p = 0.635) at 12 months (Fig 3C). Among *de novo*, restenosis, or ISR lesions, the 12-month binary restenosis-free (79%, 75%, and 80%, p = 0.456) (Fig 2D) and CD-TLR-free rates (80%, 75%, and 80%, p = 0.807) were similar (Fig 3D).

Table 4 summarizes the results of independent predictors of binary restenosis and CD-TLR identified in multivariate analysis. Lesion length >150 mm and RC 6 were identified as independent predictors for binary restenosis (Hazard ratio [HR] 8.00, 95% confidence interval [CI] 1.12–55.6, p = 0.038 and HR 7.09, 95% CI 1.15–43.5, p = 0.034, respectively). Occlusive lesions had a marginally significant higher CD-TLR rate (HR 2.38, 95% CI 0.98–5.79, p = 0.056) than stenotic lesions.

**Table 4:** Multivariate analyses: predictors of binary restenosis and clinically driven target lesion revascularization (CD-TLR).

Factors	Binary restenosis HR (95% CI)	P value	CD-TLR HR (95% CI)	P value
Age	1.05 (0.99–1.10)	0.107	1.08 (0.98–1.19)	0.130
Gender	1.79 (0.40–8.06)	0.447	1.10 (0.20–6.13)	0.916
Diabetes Mellitus	1.21 (0.29–5.08)	0.792	1.02 (0.13–7.75)	0.986
Hypertension	1.30 (0.23–7.30)	0.769	5.81 (0.45–76.9)	0.179
CAD	2.97 (0.69–12.7)	0.143	2.08 (0.49–8.88)	0.321
CHF	1.79 (0.30–10.8)	0.525	5.54 (0.48–63.9)	0.170
CVA	1.42 (0.31–6.37)	0.649	1.02 (0.12–8.60)	0.988
Dialysis	1.12 (0.18–6.82)	0.906	1.95 (0.25–15.2)	0.521
Smoking	3.11 (0.65–14.7)	0.154	3.19 (0.55–18.5)	0.195
Hyperlipidemia	2.85 (0.80–10.1)	0.105	2.49 (0.51–11.4)	0.269
Vessel calcification		0.906		0.583
Moderate *vs*. mild	1.51 (0.23–9.90)	0.668	3.31 (0.34–32.2)	0.302
Severe *vs*. mild	1.14 (0.25–5.18)	0.867	2.13 (0.33–13.8)	0.429
Occlusion	1.52 (0.72–3.24)	0.277	2.38 (0.98–5.79)	0.056
Poor runoff^¶^	1.38 (0.25–7.71)	0.711	2.18 (0.26–18.5)	0.475
Bailout stent	0.63 (0.16–2.50)	0.517	0.61 (0.10–3.80)	0.597
Rutherford class		0.180		0.375
RC class 4 *vs*. 3	3.34 (0.70–15.9)	0.129	4.07 (0.60–27.0)	0.149
RC class 5 *vs*. 3	2.93 (0.34–25.0)	0.327	5.24 (0.33–83.3)	0.240
RC class 6 *vs*. 3	7.09 (1.15–43.5)	0.034*	5.78 (0.66–50.0)	0.113
LL >150 mm	8.00 (1.12–55.6)	0.038*	7.30 (0.47–111)	0.156

Abbreviations: CAD: coronary artery disease; CHF: congestive heart failure; CVA: cerebrovascular accident; RC: Rutherford class; LL: lesion length.

^¶^ poor runoff was defined as one vessel or none of below-the-knee runoff

## Discussion

Our results demonstrated the efficacy of the DCB procedure in high-risk patients with FP disease. Despite general comorbidity and advanced limb ischemia in these patients, there were no statistical differences in terms of 1-year binary restenosis-free and CD-TLR-free rates when compared to low- and moderate-risk patients.

For patients with symptomatic FP disease, bare metal nitinol stents have reduced 12-month restenosis rates to 20–40% for lesions of <10 cm in randomized controlled trials [2, 16]. However, currently available endovascular options, including plain old balloon angioplasty, subintimal angioplasty, and provisional or primary nitinol stenting, are still limited in their long-term durability for TASCII C/D lesions [8, 17–18]. The STELLA study reported 1-year primary patency rates as 82.1% and 44% for TASC II C and D lesions, respectively [19]. The average lesion and stented lengths were 220 ± 160 mm and 260 ± 180 mm, respectively.

Promising results using DCBs to treat shorter lesions are well established [9–12], but the outcomes of DCBs for long FP lesions are sparse. In our study, results of DCBs in TASC II B lesions are similar to those reported in previous DCB proof-of-concept trials, with 10% binary restenosis and 5% CD-TLR rates at 12 months. Half of the patients in this subgroup had tissue loss, and the stent crossover rate was only 10%. For TASC II C/D lesions, the 12-month binary restenosis and CD-TLR rates were 29% and 20%, respectively. Our results are consistent with those reported by Zeller et al. of 23.9% and 15.6% 12-month binary restenosis and CD-TLR rates following the DCB procedure for long *de novo* or restenotic lesions (mean LL, 194 ± 86 mm) [13]. The provisional stenting rate in our study (30%) was higher than those reported by Zeller (18.3%). We assumed the higher stenting rate might be associated with other comorbidities, and calcified lesions in more dialyzed patients in this registry.

Lesion length has commonly been found as an independent factor for restenosis under currently available EVT [1, 20–21]. Although our study showed promising results with DCBs, as well as, a reduction in the number of implanted stents in long FP disease, lesion length >150 mm remained an independent factor for binary restenosis in multivariate analysis. Nevertheless, the application of DCBs in longer length lesions requires further large-scale investigation.

Diffuse ISR or in-stent occlusion remains a troublesome scenario to treat [15, 22, 23], with recurrent restenosis rates > 70% at 1 to 2 years. DCBs have been used to treat FP ISR (mean LL < 150 mm) with some success [24, 25]. In our study, all patients presented with type II or type III ISR, with a mean LL 253 ± 129 mm. Although the number of patients with ISR was small, the 1-year binary restenosis- and CD-TLR-free rates were 80% and 80%, respectively. Furthermore, the 1-year CD-TLR rate did not differ between type II and type III ISR lesions (13% *vs*. 33%, p = 0.347). This result suggested that DCBs might be valuable in treating diffuse ISR or in-stent occlusions.

In comparison to the encouraging results of DCBs for relatively simple lesions, very few high-risk (CLI or dialysis-dependent) patients are enrolled into randomized trials (6–16%). Therefore, reliable information regarding the appropriate application of DCBs in these patients is not currently available. In our study, we included the largest number of CLI and dialysis patients to be reported in the literature and used the FeDCLIP score for risk stratification, which has been indicated to predict vessel patency and mortality in different risk patients following self-expanding nitinol stent intervention in FP disease. Soga et al. reported the 1-year primary patency as 53% for high-risk patients undergoing FP stenting [14]. This result seemed lower than in our high-risk patients receiving the DCB treatment (73% primary patency). Local drug delivery via the paclitaxel-coated balloon did not display significant discrepancy with regards to vessel patency and CD-TLR in each risk group. Although, the effects of antiproliferative coating on wound healing, and limb salvage and survival rates, remain unclear and need to be further investigated. Outcomes of the DCB procedure in high-risk and critically ill patients seemed to be satisfactory. However, RC 6 remained an independent risk factor for binary restenosis. Extreme atherosclerosis in these patients and higher vascular inflammation during the index procedure (mean CRP levels in RC 6 *vs*. RC 5 *vs*. RC 4: 12.55 *vs*. 3.56 *vs*. 2.46 mg/dL, p = 0.027) might have negatively influenced the DCB outcome in these patients.

One target vessel thrombosis was noted in this study, a historic concern for local drug delivery; however, this event was not related to a drug effect. IVUS during reintervention confirmed that recoil of subintimal and lack of bailout stenting reduced flow and caused subsequent arterial occlusion.

This study also has several limitations that remain to be addressed. First, this is a retrospective observational analysis with a small sample size and short follow-up time. In addition, wide use of DCBs for all patients with peripheral artery disease was not plausible due to cost and lack of provisions for reimbursement via health insurance. Second, single-institution series are often biased towards particular patient demographics and practice patterns, but these data represent the real-world application of DCBs in high-risk patients with FP disease. Third, IVUS and follow-up angiography were not routinely performed, thus the detailed late lumen loss were unavailable. Lastly, the severity of calcium burden was assessed by fluoroscope without axial CT imaging. A previous study reported that circumferential calcium distribution might have a significant impact on the DCB efficacy [26]. Therefore, patients with severe calcification were pretreated with debulking or plaque modification devices before using the DCB, which might attenuate the impact of calcium burden on long-term outcomes.

In conclusion, 1-year outcomes of the DCB treatment in high-risk patients with FP disease were effective, despite general comorbidities and advanced limb ischemia. The DCB procedure holds promise to improve vessel patency, however, lesion length >150 mm and major tissue loss were identified as independent predictors for binary restenosis after the treatment.
